# Sirt1 carboxyl-domain is an ATP-repressible domain that is transferrable to other proteins

**DOI:** 10.1038/ncomms15560

**Published:** 2017-05-15

**Authors:** Hyeog Kang, Shinichi Oka, Duck-Yeon Lee, Junhong Park, Angel M. Aponte, Young-Sang Jung, Jacob Bitterman, Peiyong Zhai, Yi He, Hamed Kooshapur, Rodolfo Ghirlando, Nico Tjandra, Sean B. Lee, Myung K. Kim, Junichi Sadoshima, Jay H. Chung

**Affiliations:** 1 Laboratory of Obesity and Aging Research, Genetics and Development Biology Center, National Heart Lung and Blood Institute, National Institutes of Health, Bethesda, Maryland 20892, USA; 2 Department of Cell Biology and Molecular Medicine, Cardiovascular Research Institute, Rutgers University, New Jersey Medical School, Newark, New Jersey 07101, USA; 3 Biochemistry Core Facility, Biochemistry and Biophysics Center, National Heart, Lung and Blood Institute, National Institutes of Health, Bethesda, Maryland 20892, USA; 4 Tulane University School of Medicine, Department of Pathology, New Orleans, Louisiana 70112, USA; 5 Proteomics Core Facility, National Heart Lung and Blood Institute, National Institutes of Health, Bethesda, Maryland 20892, USA; 6 Integrated Metabolomics Research Group, Western Seoul Center, Korea Basic Science Institute, Seoul 120-140, Republic of Korea; 7 Laboratory of Molecular Biophysics, National Heart Lung and Blood Institute, National Institutes of Health, Bethesda, Maryland 20892, USA; 8 Laboratory of Molecular Biology, National Institute of Diabetes and Digestive and Kidney Diseases, National Institutes of Health, Bethesda, Maryland 20892, USA

## Abstract

Sirt1 is an NAD^+^-dependent protein deacetylase that regulates many physiological functions, including stress resistance, adipogenesis, cell senescence and energy production. Sirt1 can be activated by energy deprivation, but the mechanism is poorly understood. Here, we report that Sirt1 is negatively regulated by ATP, which binds to the C-terminal domain (CTD) of Sirt1. ATP suppresses Sirt1 activity by impairing the CTD's ability to bind to the deacetylase domain as well as its ability to function as the substrate recruitment site. ATP, but not NAD^+^, causes a conformational shift to a less compact structure. Mutations that prevent ATP binding increase Sirt1's ability to promote stress resistance and inhibit adipogenesis under high-ATP conditions. Interestingly, the CTD can be attached to other proteins, thereby converting them into energy-regulated proteins. These discoveries provide insight into how extreme energy deprivation can impact Sirt1 activity and underscore the complex nature of Sirt1 structure and regulation.

## Abstract (web summary)

The deacetylase Sirt1, known to regulate many cellular functions, can be activated by energy deprivation, however the mechanism is unclear. Here, the authors show that ATP inhibits Sirt1 by binding to the C-terminal domain, and energy deprivation derepresses Sirt1 activity by lowering the ATP level.

Sirtuins are a family of conserved NAD^+^-dependent deacetylases that play important regulatory roles in diverse cellular processes^1^. Sirtuins deacetylate modified lysine residues in protein substrates, converting NAD^+^ into nicotinamide and 2′-*O*-acetyl-ADP-ribose. The founding member of the Sirtuins is yeast Sir2 (ref. 2), and Sirt1 is the closest mammalian ortholog^3^,^4^. Sirt1 deacetylates a number of substrates, including NF-κB (refs 5, 6, 7), p53 (refs 8, 9, 10, 11), p300 (ref. 12), PGC-1α (refs 13, 14) and FOXO^15^,^16^,^17^ transcription factors and regulates a wide array of functions ranging from stress resistance^8^,^9^,^10^,^11^,^15^,^18^,^19^ to adipogenesis^20^,^21^.

Sirt1 deacetylase activity can be regulated by interactions with cellular proteins^22^,^23^,^24^,^25^, the best characterized being DBC1 (Deleted in Breast Cancer 1), which inhibits Sirt1 activity by binding to the deacetylase (catalytic) domain of Sirt1 (refs 26, 27). The Sirt1-DBC interaction is dynamically regulated. Genotoxic stress enhances it via ATM-mediated phosphorylation of Sirt1 (ref. 28). Conversely, energy starvation^29^ and the resulting activation of the AMP-activated protein kinase (AMPK) pathway^30^,^31^,^32^ disrupt it. However, the amount of DBC1 relative to Sirt1 is low in some cell types^29^, raising the possibility a fraction of Sirt1 is not under suppression by DBC1.

The deacetylase (catalytic) domain of Sirt1, which is conserved in sirtuins, is flanked by the N-terminal domain (NTD)^33^ and C-terminal domain (CTD), both of which have regulatory functions^34^,^35^. Sirt1 is unique among sirtuins in that it has a particularly long CTD (>230 residues), the function of which is poorly understood. The CTD is largely disordered and/or flexible and contains a remote 25 residue (residues 641–665 in human Sirt1 and 631–655 in mouse Sirt1) peptide ESA (Essential for Sirt1 Activity), which is critical for Sirt1 activity^33^,^34^,^36^,^37^. ESA is not only important for Sirt1 substrate interaction^34^ but also by looping over, it interacts with and augments the β-sheet of the Rossman fold in the deacetylase domain^33^,^34^,^36^,^37^,^38^. DBC1 inhibits Sirt1 activity in part by blocking the ESA-deacetylase domain interaction^34^.

The mechanism by which energy starvation activates Sirt1 is incompletely understood. ATP is the major energy currency molecule in cells and can function as an energy indicator. For example, the ATP-sensitive K^+^ channel regulates electrical excitability in multiple tissues in response to the intracellular ATP concentration^39^. Conditions that overwhelm the homeostatic mechanisms, such as ischaemia or treatment with 2-deoxy-D-Glucose (2-DG), a non-metabolizable glucose analogue, can lead to ATP-depletion^40^. Free cytosolic concentration of ATP is extremely high under energy-charged conditions. The intracellular ATP concentration range is 1–10 mM in many cell types and is 5–11 mM in the heart^41^,^42^,^43^. With ATP depletion, concentrations of ADP and AMP transiently increase but remain in the micromolar range: 0–500 μM for ADP and and 0–50 μM for AMP. This is because serial hydrolysis of ATP→ADP→AMP→adenosine depletes them over time^41^,^42^,^43^.

Here, we investigated the possibility that severe energy depletion activates Sirt1 via ATP. We were led to consider this possibility when we discovered that severe energy depletion increases Sirt1 activity despite the decrease of NAD^+^, which is dependent on ATP for *de novo* synthesis, and it occurred independently of AMPK. In the physiological concentration range, ATP binds to and represses ESA function and Sirt1 activity, and depletion of ATP increases Sirt1 interaction with its substrates and its deacetylase activity. Mutations that interfere with ATP binding increase Sirt1's ability to resist stress and inhibit adipogenesis under energy-charged conditions. The CTD can be attached to unrelated proteins and confer on them the ability to bind Sirt1 substrates in an energy-sensitive manner.

## Results

### ATP inhibits Sirt1 activity

**Figure 1:**
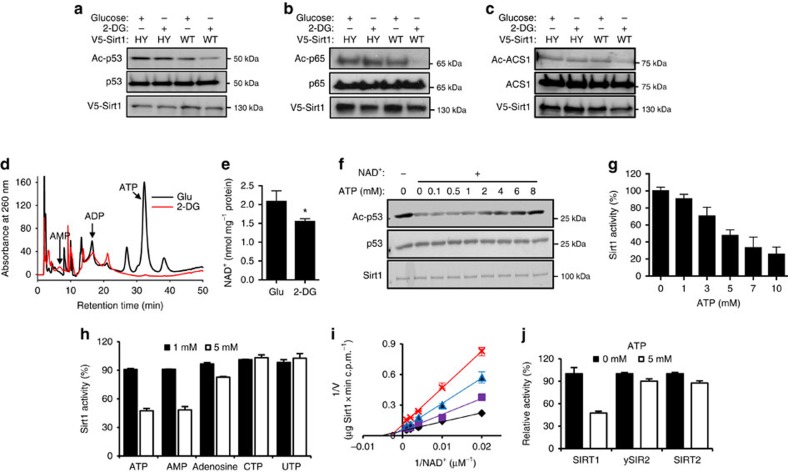
ATP inhibits Sirt1 activity. (**a**) Treatment with 2-deoxyglucose (2-DG) activates Sirt1. 293 HEK cells were co-transfected with the expression vectors for p53 and either HY or WT Sirt1 as indicated. Twenty-four hours later, each plate was divided into two plates and further cultured. Forty-eight hours after transfection, cells were incubated with either 25 mM Glucose (Glu) or 25 mM 2-DG for 2 h. The acetylation status of K382 in p53 was evaluated by immunoblotting with an antibody specific for acetylated K382. The experiment shown in **a** was repeated using FLAG-tagged p65 protein (a component of NF-κB) (**b**) and FLAG-tagged acetyl CoA synthestase1 (ACS1) (**c**). The acetylation of p65 and ACS1 were assessed by immunoprecipitation with FLAG antibody followed by immunoblotting with antibody specific for acetylated K310 for p65 and acetylated lysine antibody for acetylated ACS1. (**d**) Cells were treated with media containing either 25 mM Glu or 2-DG for 2 h and the intracellular levels of ATP, ADP and AMP were measured by HPLC. (**e**) The intracellular levels of NAD^+^ after Glu or 2-DG treatment were determined by HPLC (*n*=4). The HPLC chromatogram is shown in Supplementary Fig. 1a. (**f**) ATP inhibits Sirt1 activity. Deacetylation of Ac-p53 by recombinant Sirt1 in the presence of 0–8 mM ATP. Deacetylation in the absence of NAD^+^ is shown as a negative control. (**g**) Sirt1 activity against Ac-H4 as a function of ATP (0–10 mM) (*n*=4). Results are presented as % of control (0 mM ATP). (**h**) Sirt1 activity against Ac-H4 in the presence of either 1 or 5 mM of ATP, AMP, adenosine, CTP and UTP as a percentage of the Sirt1 activity in the absence of the nucleotides (*n*=4). (**i**) Lineweaver–Burk plot of recombinant Sirt1 activity in the presence of 0 (⧫), 4 (✓), 6 (▴) and 8 (× ) mM ATP for the range of NAD^+^ (*n*=3). (**j**) The activities of yeast Sir2 and Sirt2 are relatively resistant to ATP. The relative activities of Sirt1, yeast Sir2 and Sirt2 against Ac-H4 in the presence of 0 or 5 mM ATP is shown (*n*=4).

To investigate how severe energy depletion affects Sirt1 activity, we replaced glucose (25 mM) with 2-DG (25 mM) rather than adding 2-DG to the glucose-containing media^44^. Two hours later, we visualized deacetylation of three Sirt1 substrates, p53 (K382) (Fig. 1a), p65 (NF-κB) (Fig. 1b) and acetyl CoA synthestase1 (ACS1; Fig. 1c) in cells transiently expressing wild-type (WT) Sirt1 and catalytically inactive H355Y Sirt1 (HY). For all the experiments shown in this paper, we used the mouse Sirt1 expression vector. Treatment with 2-DG increased deacetylation of p53, p65 and ACS1 in the presence of WT Sirt1, but not in the presence of HY Sirt1, indicating that Sirt1 is activated by 2-DG. Under these conditions, levels of ATP decreased dramatically (Fig. 1d) and levels of NAD^+^ decreased modestly (Fig. 1e and Supplementary Fig. 1a). To rule out the possibility that 2-DG increased Sirt1 activity indirectly by activating AMPK^30^,^31^, which is known to be activated by 2-DG (ref. 45), we treated WT and AMPK-deficient murine embryo fibroblasts (MEFS) with 2-DG (Supplementary Fig. 1b). We found that 2-DG increased p53 deacetylation even in AMPK-deficient MEFS, indicating that 2-DG increased Sirt1 activity in an AMPK-independent manner.

The findings in Fig. 1a–e led us to test the possibility that Sirt1 activity can be regulated directly by ATP. To investigate the possible effect of ATP on Sirt1 activity, we performed catalytic reactions with recombinant Sirt1 using p53 acetylated by p300 as substrate in the presence of 0–8 mM ATP (with Mg^2+^). As shown in Fig. 1f, deacetylation of K382 in p53, which is mediated by Sirt1, is suppressed by ATP, starting from ≥1 mM concentration. Compared to ATP, GTP did not significantly inhibit Sirt1 (Supplementary Fig. 1c). Consistent with this, measurement of Sirt1 initial velocity using [^3^H]-labelled histone H4 also showed that ATP decreased Sirt1 activity in a concentration-dependent manner (Fig. 1g). In contrast, adenosine, CTP and UTP suppressed Sirt1 activity poorly, if at all (Fig. 1h). Interestingly, at 1 and 5 mM concentrations, AMP and ATP showed similar inhibition of Sirt1 activity. However, AMP and ADP do not reach millimolar concentration *in vivo* due to the activities of 5′-nucleotidase and adenylate kinase, respectively (see Fig. 1d and refs 41, 42, 43).

Since NAD^+^ has the adenosine moiety in it, ATP may be inhibiting Sirt1 by simply competing with NAD^+^. To examine this possibility, we performed catalytic reactions with varying concentrations of NAD^+^ and ATP to generate a Lineweaver–Burk plot (Fig. 1i), which indicated that ATP is a non-competitive inhibitor against NAD^+^. To further confirm that ATP is not a competitive inhibitor of NAD^+^, we performed catalytic reactions with yeast Sir2 (ySir2) and Sirt2 (refs 46, 47), which are also NAD^+^-dependent, in the presence of ATP. As shown in Fig. 1j, neither ySir2 nor Sirt2 were inhibited significantly by ATP, indicating that ATP-sensitivity is not a general property of sirtuins.

### ATP binds to the CTD of Sirt1

**Figure 2:**
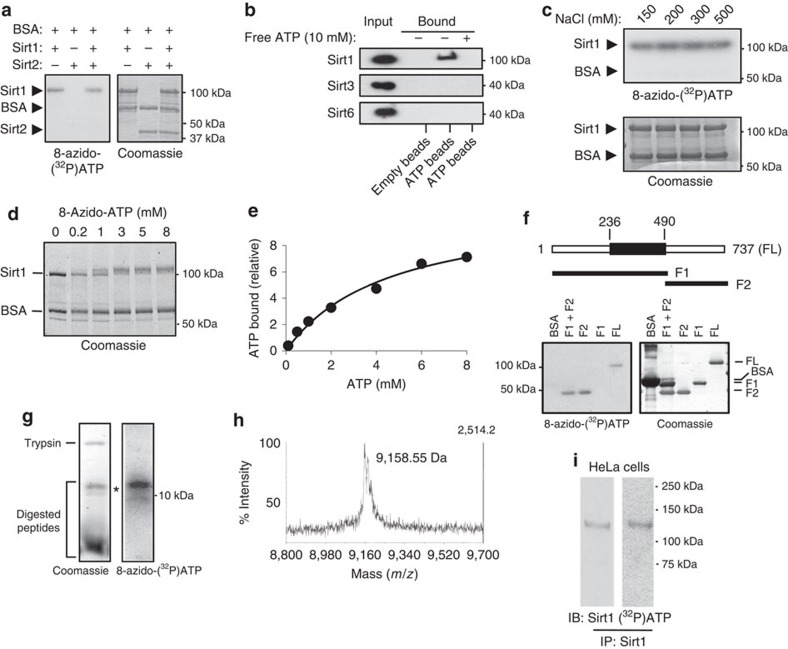
ATP binds to the CTD of Sirt1. (**a**) Photoaffinity-labelling of recombinant Sirt1, BSA and recombinant Sirt2. Photoaffinity-labelling of 8-azido-[α-^32^P]ATP was visualized by autoradiography (left panel) and total protein levels were visualized by Coomassie staining (right panel). (**b**) His-tagged Sirt1, Sirt3 or Sirt6 were incubated with empty agarose beads or ATP-conjugated agarose beads in the absence or presence of 10 mM ATP for 4 h. Bound proteins were eluted by SDS sample buffer and visualized by immunoblotting with a His-tag specific antibody. (**c**) Photoaffinity-labelling of recombinant Sirt1 and BSA with 8-azido-[α-^32^P]ATP in the presence of 150–500 mM NaCl. Photoaffinity-labelling of 8-azido-[α-^32^P]ATP was visualized by autoradiography (top panel) and total protein levels were visualized by Coomassie staining (bottom panel). (**d**) Visualization of ATP-binding by mobility-shift. Sirt1 and BSA were photoaffinity-labelled with 0–8 mM of 8-azido-ATP, electrophoresed in SDS–PAGE and visualized by Coomassie staining. (**e**) The concentration dependence of ATP binding to Sirt1. ATP binding was measured by using the gel filtration technique and *K*_d_ was calculated to be ∼4.6 mM (*n*=3). (**f**) A schematic diagram of mouse Sirt1 fragments. The deacetylase domain is shown as a black rectangle. The CTD of Sirt1 binds to ATP. Photoaffinity-labelling of fragments F1, F2 and BSA with 8-azido-[α-^32^P]ATP is shown. (**g**) Tryptic digest of Sirt1 photoaffinity-labelled with 8-azido-[α-^32^P]ATP was electrophoresed in 20% SDS–PAGE. The peptide labelled with 8-azido-[α-^32^P]ATP was visualized with autoradiography (right) and all of the peptides were visualized by Coomassie staining (left). (**h**) The mass of the photoaffinity-labelled peptide (*, **g**) was determined by MS/MS. The absolute intensity of the peak is indicated on the right *Y* axis. The sequence analysis of the peptide is shown in Supplementary Table 1. (**i**) Endogenous Sirt1 also binds ATP. Permeabilized HeLa cells were incubated with [α-^32^P]ATP and endogenous Sirt1 was immunoprecipitated after photoaffinity-labelling with ultraviolet radiation. Endogenous Sirt1 labelled with [α-^32^P]ATP was detected by autoradiography (right) and immunoblotting with anti-Sirt1 antibody (left).

To better characterize the Sirt1-ATP interaction, we incubated Sirt1, Sirt2 and bovine serum albumin (BSA) with 8-azido-[α^32^P]ATP, an ATP analogue that crosslinks to ATP-binding proteins when exposed to ultraviolet radiation^48^. As shown in Fig. 2a, 8-azido-[α^32^P]ATP photoaffinity-labelled Sirt1 but not BSA or Sirt2. We also incubated recombinant Sirt1, Sirt3 and Sirt6 with ATP-conjugated beads or empty beads and examined their binding to ATP (Fig. 2b). Among them, only Sirt1 bound to ATP beads, and competing free ATP prevented its binding to ATP beads. The Sirt1-ATP interaction was not diminished even at a very high NaCl concentration (500 mM), indicating that ATP binding is not due to nonspecific charge interactions (Fig. 2c). To examine whether ATP-binding affected the electrophoretic mobility of Sirt1, we photoaffinity-labelled Sirt1 and BSA with 8-azido-ATP and analysed them by SDS–polyacrylamide gel electrophoresis (SDS–PAGE; Fig. 2d). The electrophoretic mobility of Sirt1, but not of BSA, was retarded in a concentration-dependent manner by 8-azido-ATP-binding. Next, we measured Sirt1's affinity for ATP. For this purpose we employed gel-filtration using [^32^P]ATP. The ATP-binding curve indicated that Sirt1 had a dissociation constant (*K*_d_) of ∼4.6 mM (Fig. 2e), which is close to the IC_50_ of 4–5 mM (Fig. 1f) and within the dynamic range of physiological ATP concentration.

To identify the ATP-binding region in Sirt1, we divided mouse Sirt1 into two regions: F1 (a.a. 1–490), which contains the N-terminal domain and the deacetylase domain and F2 (a.a. 491–737), which contains the CTD (Fig. 2f). These two fragments, along with full-length Sirt1 (FL), were photoaffinity-labelled with 8-azido-[α^32^P]ATP and visualized by autoradiography after separation in SDS–PAGE. We found that FL and F2 were strongly labelled, but F1 and BSA were not labelled, indicating that the CTD is the major ATP binding region.

To narrow down the ATP-binding site, we photoaffinity-labelled full-length mouse Sirt1 with 8-azido-[α^32^P]ATP and digested the complex with trypsin before separating the mixture in 20% SDS–PAGE. As shown in Fig. 2g, digestion with trypsin generated many peptide bands, but only the largest peptide band (∼10 kD) was strongly labelled. This band was excised from the gel and was identified by using LTQ-Orbitrap MS/MS (Fig. 2h) to be the 85 a.a. fragment (residues 640–724 in mouse Sirt1) in the CTD (Supplementary Table 1).

A caveat to these experiment is that the recombinant Sirt1 produced in *Escherichia coli* is likely to have post-translational modifications that are different from that produced in mammalian cells and 8-azido-ATP, which is routinely used as an ATP substitute for ATP-binding studies, may have physicochemical properties that are different from ATP. Therefore, we tested whether endogenous cellular Sirt1 could also bind to ATP. For this purpose, we incubated [α^32^P]ATP, which can photoaffinity-label ATP-binding proteins, albeit not as efficiently as 8-azido-[α^32^P]ATP, with permeabilized HeLa cells and exposed them to ultraviolet radiation. Endogenous Sirt1 was then immunoprecipitated and electrophoresed by using SDS–PAGE (Fig. 2i). A band co-migrating with Sirt1 was labelled with [α^32^P]ATP suggesting that endogenous Sirt1 also bound ATP.

### ATP interferes with CTD function

Interestingly, the ATP-binding peptide partially overlaps with the ESA (residues 631–655 in mouse Sirt1). Structural studies of adenine nucleotide-binding proteins indicate that they bind to adenine nucleotides through hydrogen and ionic bonds involving numerous polar and charged amino acid residues^49^. To identify the residues that are important for ATP binding we mutated seven polar or positively charged residues in the ESA peptide in different combinations: Ser 649/651, Tyr 632/640/648, Asn 638 and Arg 639 (Supplementary Fig. 2a). Compared to the WT ESA peptide, the ESA peptide containing 2A, 3A and 4A mutations had reduced ATP-binding and the 7A mutation almost abolished ATP-binding (Supplementary Fig. 2b). The serine residues mutated in 2A (S649 and S651) are two of the four serine residues phosphorylated by CK2 in response to ionizing radiation^50^. Phosphorylating these two serines (2P, Supplementary Fig. 2b) in the ESA peptide also decreased its ATP binding. To determine if the 7A mutation also affected Sirt1 activity, we performed catalytic reactions using WT or 7A Sirt1 or ΔESA Sirt1, which is missing the ESA region. As shown in Supplementary Figs 2c and 2d, 7A Sirt1, like ΔESA Sirt1, had no catalytic activity *in vitro* and *in vivo*, respectively.

**Figure 3:**
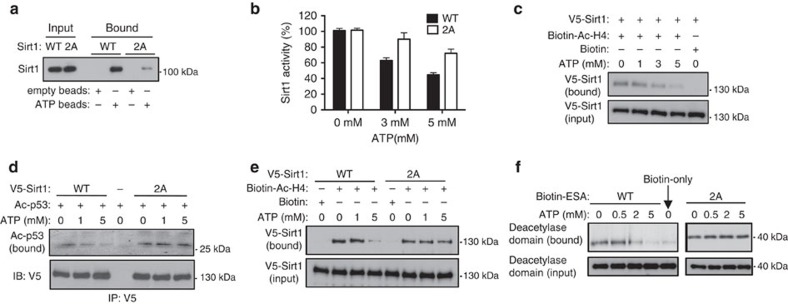
ATP interferes with CTD function. (**a**) His-tagged WT or 2A mutant Sirt1 was incubated with either agarose or ATP-agarose beads and bound Sirt1 was visualized by immunoblotting. (**b**) The catalytic activity of recombinant 2A mutant Sirt1 is less sensitive to ATP. The deacetylation of Ac-H4 by recombinant WT or 2A mutant Sirt1 in the presence of 0–5 mM ATP (*n*=6). Comparisons between the treatment groups were analysed by two-tailed unpaired Student's *t*-test. (**c**) ATP reduces Sirt1-substrate interaction. Biotinylated Ac-H4 peptide was immobilized to streptavidin beads and incubated with V5-tagged Sirt1 in the presence of the indicated concentrations of ATP. Substrate-bound V5-tagged Sirt1 was visualized by immunoblotting with anti-V5 antibody. Biotin immobilized to streptavidin beads without Ac-H4 was used as a negative control. (**d**) Ac-p53 interaction with 2A Sirt1 is less sensitive to ATP than with WT Sirt1. After incubating V5-tagged WT or 2A Sirt1 with Ac-p53 in the presence of the indicated concentrations of ATP, Sirt1 was pulled down with anti-V5 antibody. Ac-p53 bound to either WT or 2A Sirt1 is shown. For the negative control, we incubated Ac-p53 with no Sirt1. (**e**) Ac-H4 interaction with 2A Sirt1 is less sensitive to ATP than with WT Sirt1. Ac-H4 interaction with Sirt1 with increasing concentrations of ATP was visualized as in **a** except 2A Sirt1 was also included. (**f**) The interaction between the deacetylase domain and the ESA peptide is ATP-sensitive. Deacetylase domain pull-down experiments were performed after streptavidin-immobilized WT or 2A mutant ESA peptides were incubated with recombinant deacetylase domain (a.a. 236–490) in the presence of 0–5 mM ATP. The amount of the deacetylase domain pulled down is shown. Almost no deacetylase domain was pulled down with streptavidin resin alone.

Since the 2A mutation disrupts ATP binding with the smallest number of mutated residues, we focused on the characterization of the 2A mutation. In full-length Sirt1, the 2A mutation significantly reduced Sirt1 binding to ATP beads, although not completely (Fig. 3a). Consistent with this, the inhibitory effect of ATP on Sirt1 activity was blunted with the 2A mutation (Fig. 3b). WT and 2A Sirt1 had similar *K*_m_ (26.83±2.32 μM and 23.11±0.80 μM, respectively for Ac-H4 and 203.08±13.24 μM and 186.01±7.07 μM, respectively, for NAD^+^) and *k*_cat_ (0.43±0.08 min^−1^ and 0.41±0.12 min^−1^, respectively, for acetylated-histone H4 (Ac-H4) and 0.40±0.04 min^−1^ and 0.54±0.08 min^−1^, respectively for NAD^+^; Supplementary Table 2). However, the 2A Sirt1 had higher ATP inhibition constants *K*_ii_ (6.45±0.51 versus 4.16±0.16 mM) against Ac-H4 compared to WT Sirt1 (Supplementary Table 3), indicating that 2A Sirt1 is more resistant to the inhibitory effect of ATP.

ESA interacts with the deacetylase domain and promotes interaction with Sirt1 substrates^34^. We hypothesized that ATP binding to the ESA may inhibit both the Sirt1-substrate and the ESA-deacetylase domain interactions. To test this hypothesis, we performed pull-down experiments by incubating streptavidin-immobilized substrate (biotin-Ac-H4) with recombinant Sirt1 in the presence of increasing concentrations of ATP. As shown in Fig. 3c, the amount of Sirt1 bound to the substrate decreased with increasing ATP concentration. We then asked if the 2A mutation decreased ATP's ability to suppress the Sirt1-substrate interaction. Sirt1 interaction with two substrates, Ac-p53 (Fig. 3d) and Ac-H4 (Fig. 3e), were tested. For both substrates, ATP reduced their interaction with WT Sirt1, but the 2A mutation blunted this effect. We then tested the effect of ATP on the ESA-deacetylase domain interaction by performing pull-down assays with the immobilized biotinylated-ESA peptide after incubating with the deacetylase domain in the presence of increasing ATP concentration (0–5 mM). As shown in Fig. 3f, the WT ESA-deacetylase domain interaction was inhibited by ATP in a concentration-dependent manner but the 2A mutation blunted this effect. Taken together, these findings indicate that ATP inhibits Sirt1-substrate and ESA-deacetylase domain interactions and the 2A mutation blunts this effect.

### ATP binding suppresses Sirt1 activity *in vivo*

**Figure 4:**
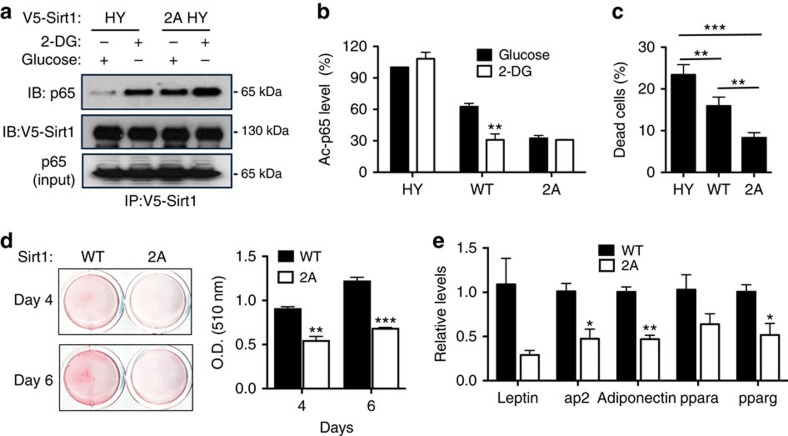
ATP binding suppresses Sirt1 activity *in vivo.* (**a**) Sirt1 interaction with substrate is regulated by ATP *in vivo*. FLAG-tagged p65 was co-expressed in 293 HEK cells with either V5-tagged HY mutant or 2A HY double mutant Sirt1. Co-immunoprecipitation was performed by immunoprecipitating with anti-V5 antibody followed by immunoblotting with either anti-FLAG antibody for detection of bound protein or anti-V5 antibody for immunoprecipitants. (**b**) Sirt1 KO MEFS stably expressing V5-tagged HY, WT or 2A Sirt1 were incubated with media containing either 25 mM Glu or 25 mM 2-DG for 2 h and acetylation of p65 was monitored by immunoblotting whole-cell extracts with acetyl p65 (K310) antibody. Four independent experiments were performed and data are represented as mean±s.e.m. (**c**) Sirt1 expressing MEFS from **b** were exposed to heat shock (42 °C, 30 min) and cell viability was determined after 24 h later by Trypan-blue exclusion assay (*n*=6). (**d**) Representative images of Oil Red O staining of cells stably expressing WT or 2A mutant Sirt1 4 and 6 days after addition of adipogenic cocktail (left). Adipocyte differentiation was quantified at 4 and 6 days by Oil Red O extraction (right) (*n*=3). (**e**) qRT-PCR analysis of adipogenic gene mRNA in differentiated WT and 2A mutant Sirt1 cells from (**d**). Three independent experiments were performed. *P<0.05; **P<0.01; ***P<0.001. two-tailed unpaired Student's *t*-test was used for statistical calculation.

To study the effect of changing intracellular ATP concentration on Sirt1-substrate interaction *in vivo*, we used the catalytically inactive HY Sirt1 to prevent any alteration in Sirt1-substrate interaction after deacetylation. HY Sirt1 interaction with p65, as visualized by co-immunoprecipitation, increased with 2-DG but 2A HY Sirt1 interaction was significantly higher in both glucose and 2-DG (Fig. 4a). Since HY Sirt1 does not interact with DBC1 (ref. 51), our findings indicate that ATP depletion increases Sirt1-p65 interaction in a DBC1-independent manner.

To demonstrate the effect of ATP-binding on Sirt1 activity *in vivo*, we restored Sirt1 activity in Sirt1-deficient MEFS with HY, WT or 2A Sirt1. We next measured Sirt1 activity by quantifying deacetylation of p65 in these MEFS exposed to glucose or 2-DG. As shown in Fig. 4b, 2-DG did not significantly decrease Ac-p65 in cells expressing HY Sirt1, but did so in cells expressing WT Sirt1. In cells expressing 2A Sirt1, levels of Ac-p65 were similarly low in either glucose or 2-DG indicating that 2A Sirt1 has higher activity than WT Sirt1 in energy-charged condition. Deacetylation of Foxo1, another Sirt1 substrate, showed a similar energy-dependent pattern (Supplementary Fig. 2d). Sirt1 increases heat shock response and protects against heat shock-induced cell death^19^. We measured cell death in MEFS 24 h after heat shock (42 °C for 30 min) in media containing 10% fetal bovine serum in the presence of glucose (Fig. 4c). Consistent with the 2A mutation increasing Sirt1 activity, MEFS expressing 2A Sirt1 had significantly lower death than MEFS expressing WT Sirt1. Sirt1 activity is also known to inhibit adipogenesis^20^. To further confirm that 2A Sirt1 has higher activity than WT Sirt1 in a high ATP condition, we differentiated the MEFS expressing WT Sirt1 and 2A Sirt1 into adipocytes. Indeed, 2A Sirt1 decreased adipogenesis as shown by Oil-Red-O staining, which stains lipids (Fig. 4d) and by expression of adipocyte-specific genes (Fig. 4e). Previously, it was shown that Sirt1 protects cardiac myocytes against ischaemia-reperfusion, which causes cell death largely through the production of oxygen radicals during reperfusion^52^. To determine if Sirt1 plays any role in stress resistance to severe ATP-depletion, we induced cardiac ischaemia without reperfusion in mice by constricting the left anterior descending coronary artery, which decreased the cardiac NAD^+^ and ATP levels in 1 h (Supplementary Fig. 3a). We induced ischaemia in WT and heart-specific Sirt1 knockout mice and quantified myocardial infarction (Supplementary Fig. 3b,c). WT hearts had significantly reduced myocardial infarction (pale discoloration) than Sirt1 KO hearts, suggesting that Sirt1 is cytoprotective against the stress induced by ATP depletion.

### ATP opens up Sirt1 conformation

**Figure 5:**
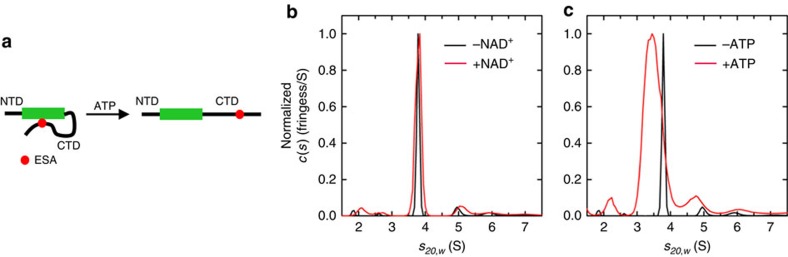
ATP opens up Sirt1 conformation. (**a**) Schematic illustration shows that ATP disrupts the ESA-deacetylase domain interaction leading to a more extended form. NTD, deacetylase domain (green box), CTD and ESA are shown. (**b**,**c**) Sedimentation velocity analysis of purified recombinant full-length Sirt1 protein. Normalized interference c(*s*) profiles for solutions of Sirt1 with 20 mM MgCl_2_ and either (**b**) 10 mM NAD^+^ or (**c**) 10 mM ATP.

The ESA-deacetylation domain interaction, which loops the CTD, is expected to make the Sirt1 conformation more compact (Fig. 5a). We hypothesized that ATP, by blocking the ESA-deacetylase interaction, and therefore CTD looping, may result in Sirt1 with a more extended conformation. To confirm this, we determined the sedimentation coefficient of Sirt1 by analytical ultracentrifugation. In the absence of any nucleotides, a major species is observed at 3.79S, consistent with the presence of a Sirt1 monomer. Addition of 10 mM NAD^+^ results in an identical sedimentation coefficient of 3.79S (Fig. 5b). However, addition of 10 mM ATP results in a broader peak with lower *s* values, indicating that ATP binding leads to a more extended conformation (Fig. 5c).

### Sirt1 CTD confers energy-sensitivity to other proteins

**Figure 6:**
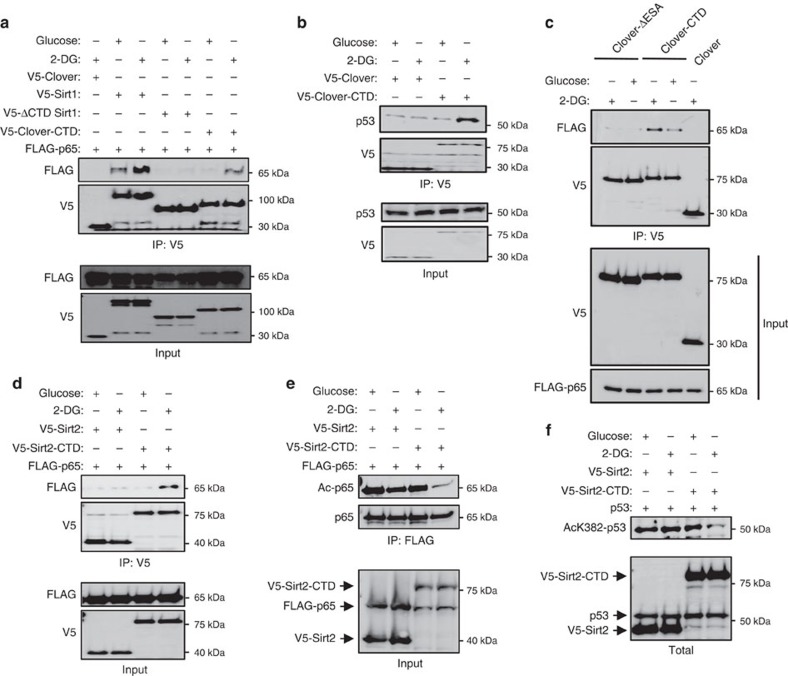
Sirt1 CTD confers energy sensitivity to other proteins. (**a**) FLAG-tagged p65 was transiently co-expressed with V5-tagged full-length Sirt1, truncated Sirt1 (ΔCTD Sirt1), Clover or Clover-CTD fusion proteins into 293 HEK cells as indicated. Forty-eight hours post transfection, cells were incubated with either 25 mM Glu or 25 mM 2-DG as indicated for 2 h. Cell extracts were immunoprecipited with anti-V5 antibody and then immunoblotted with either anti-FLAG for detection of the amount of p65 or anti-V5 for immunoprecipitants. ΔCTD and CTD indicate the Sirt1 fragments that span from 1 to 510 amino acids and from 510 to 737 amino acids of mouse Sirt1, respectively. (**b**) The experiment performed in **a** was repeated after transfections of V5-tagged Clover and Clover-CTD expression vectors into 293 HEK cells. Cells were treated for 2h with either 25 mM Glu or 25 mM 2-DG as indicated, 48 h post transfection. The binding amount of p53 with Clover or Clover-CTD was visualized by immunoprecipitating with anti-V5 antibody and immunoblotting with p53 antibody. (**c**) FLAG-tagged p65 was transiently co-expressed with V5-tagged Clover, Clover-CTD or Clover-ΔESA and co-immunoprecipitation was performed after treatment with either Glu or 2-DG as in **a**. ΔESA indicates CTD missing the ESA region (amino acids from 631 to 655). (**d**) V5-tagged Sirt2 or Sirt2-CTD was transiently co-expressed with FLAG-tagged p65 and co-immunoprecipitation was performed as above after treatment with either Glu or 2-DG. Sirt2-CTD indicates the CTD of Sirt1 is linked to the end of C-terminal end of full-length of Sirt2 as a fusion protein. (**e**,**f**) To visualize the effect of Sirt1 CTD has on deacetylase activity of Sirt2, Sirt2 or Sirt2-CTD was co-expressed with either (**e**) FLAG-tagged p65 or (**f**) p53 and treated with either Glu or 2-DG. Acetylated p65 was visualized by immunoprecipitation of p65 using followed by immunoblotting with anti-acetyl p65 (K310) antibody. Acetylated p53 level was visualized by immunoblotting with anti-acetyl-p53 (K382) antibody.

To clarify the potential role of the ESA/CTD in substrate interaction, we attached the CTD to an unrelated protein, Clover, a yellow-green fluorescent protein (Clover-CTD)^53^. As shown in Fig. 6a, the interaction of WT Sirt1 and p65 increased with 2-DG, but ΔCTD Sirt1, which is missing the CTD, interacted very poorly with p65 in both glucose and 2-DG. The interaction of Clover-CTD with p65 increased in 2-DG, whereas Clover alone did not interact with p65 at all in 2-DG. As shown in Fig. 6b, Clover-CTD interaction with p53 was also increased with 2-DG. Deleting ESA from Clover-CTD (Clover-ΔESA) abolished interaction with p65 in both glucose and 2-DG (Fig. 6c). This indicates that the ESA/CTD by itself can interact with a substrate in an ATP-sensitive manner.

If the CTD can confer substrate-binding to Clover in an ATP-sensitive manner, it may be able to convert Sirt2, which has different substrate specificity than Sirt1 and is not ATP-sensitive (Fig. 1j), into an energy-sensitive deacetylase for Sirt1 substrates. We transiently expressed Sirt2 or Sirt2-CTD, in which the Sirt1 CTD was fused to the C-terminal end of Sirt2 and visualized its interaction with p65 (Fig. 6d). Sirt2 did not interact with p65 in either glucose or 2-DG, but Sirt2-CTD interacted with p65 in 2-DG but poorly in glucose. Consistent with this, deacetylase activity of Sirt2-CTD, but not Sirt2, increased against Sirt1 substrates p65 (Fig. 6e) and p53 (Fig. 6f) in the presence of 2-DG. Taken together, these findings indicate ESA/CTD may be able to confer energy-sensitivity to other proteins.

## Discussion

Our finding that ATP, at physiological concentration range, inhibits Sirt1 suggests that Sirt1 activity is linked to the energy-charge state of the cell. The primary site of ATP-binding is the ESA in the CTD of Sirt1, which is critical for Sirt1 catalytic activity. The ESA/CTD promotes Sirt1 activity in two ways: firstly, it loops over and augments the β-sheet of the Rossman fold in the deacetylase domain (allosteric effect)^33^,^34^,^35^,^36^,^37^,^38^; secondly, it increases Sirt1 affinity for its substrates^34^. ATP appears to suppress Sirt1 activity by decreasing both of these functions of ESA/CTD (Figs 4 and 6). Our findings indicate that without ATP, ESA binds to the deacetylase domain to form an intramolecular loop, which results in a closed conformation. ATP, which inhibits the binding of ESA to deacetylase domain, opens up Sirt1 conformation (Fig. 5). In contrast, NAD^+^ has no effect on Sirt1 conformation. Mutation of S649 and S651, which decreases ATP-binding, makes Sirt1 less sensitive to ATP-mediated inhibition in the energy-charged state (Fig. 4).

Here, we showed that energy deprivation increases the deacetylation of a number of Sirt1 substrates: p53, p65, ACS1 and Foxo1. However, we do not know whether deacetylation of all Sirt1 substrates follows such energy dependence. Although we attempted to examine the deacetylation of autophagy proteins such as ATG5 and ATG7 (ref. 54) in response to energy deprivation, the basal acetylation level of these proteins were too low for us to detect deacetylation in the presence of energy deprivation.

**Figure 7:**
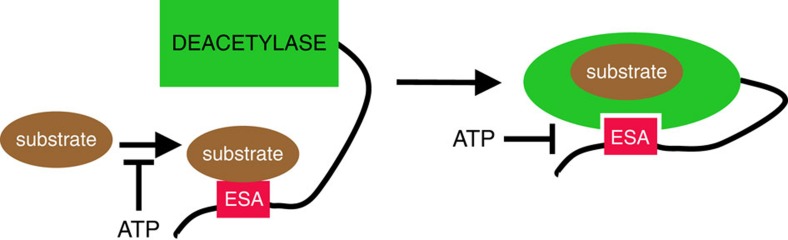
A schematic diagram of Sirt1 regulation by ATP. The ability of the ESA/CTD to both recruit substrates (left) and to allosterically activate the catalytic domain (right) is inhibited by ATP.

Since the ESA/CTD cannot increase Sirt1 activity by binding simultaneously to both the substrate and the catalytic domain (allosteric activation), we speculate that these interactions may occur sequentially (Fig. 7). We propose that the ESA/CTD, by acting as a substrate recruitment site, increases the local concentration of the substrate. This process is further complemented by the ESA allosterically activating the catalytic domain. Our findings suggest that ATP inhibits both of these steps. However, we do not fully understand the interplay between substrate recognition and ATP-binding by the ESA/CTD; addressing this question which will require further structural studies.

It should be noted that ATP is not a strong Sirt1 inhibitor: at 10 mM, it inhibits Sirt1 activity by ∼70% (Fig. 1). Furthermore, due to intracellular buffering capacity, ATP level is generally not dramatically reduced by modest energy deprivation. This suggests that Sirt1 is in a partially repressed state in energy-charged condition and is derepressed in severe energy deprivation conditions such as ischaemia, thereby increasing stress resistance and cell survival under these conditions (Supplementary Fig. 3). Unlike ATP, AMP exists in the micromolar range and is significantly more sensitive to the energy status of the cell. Thus, AMPK, which is activated by elevated AMP/ATP ratio, can increase Sirt1 activity by disrupting the Sirt1-DBC1 interaction even under modest energy deprivation^30^,^31^,^32^. We believe that the inhibitory effect of ATP is AMPK and DBC1-independent for several reasons: (1) 2-DG activates Sirt1 in AMPK-deficient MEFS (Supplementary Fig. 1b); (2) substrate interaction of inactive Sirt1 (HY), which does not bind to DBC1 (ref. 51), increases with ATP-depletion (Fig. 4a); (3) the CTD confers energy-sensitivity to Clover and Sirt2 (Fig. 6), which are not regulated by DBC1 (ref. 26). On the basis of these properties, we propose that that ATP- and AMPK-mediated regulations make up two layers of energy-sensitive response of Sirt1, each responding to different levels of energy-deprivation severity.

Attaching the Sirt1 CTD to Sirt2, which does not have ESA and is not sensitive to ATP, makes its activity and the ability to bind protein substrates energy-sensitive (Fig. 6). It can also be attached to a completely unrelated protein such as Clover, which makes its interaction with a Sirt1 substrate energy-sensitive. To the best of our knowledge, this is the first example of energy-sensitive module that can be transferred to unrelated proteins. It would be interesting to speculate that the disordered nature of the CTD makes this possible. On the basis of these findings, we believe that the CTD is a stand-alone ATP-sensitive substrate recruitment site.

These observations highlight the complexity of Sirt1 regulation that had not been fully appreciated previously. Since Sirt1 activity increases mitochondrial function^55^,^56^,^57^ and ATP production (Supplementary Fig. 3d), the inhibitory effect of ATP on Sirt1 activity suggests a negative feedback loop for maintaining energy homeostasis. Since modest energy deprivation does not significantly alter intracellular ATP concentration, the role of ATP in Sirt1 regulation may be reserved for conditions that lead to severe energy deprivation that require a particularly strong stress response.

## Methods

### Plasmids and protein purification

Recombinant WT and 2A Sirt1(mouse), F1 fragment (amino acid residues 1–490 of mouse Sirt1), F2 fragment (amino acid residues 491–734 of mouse Sirt1) and deacetylase core (amino acid residues 184–510 of mouse Sirt1) were constructed in the pET15b prokaryotic expression vector by using the Nde1 and Xho1 sites. Prokaryotic expression vectors for human Sirt2, Sirt3 and Sirt6 were kind gift from Dr John M Denu (University of Wisconsin). They were expressed in *E. coli*, and purified on Ni-NTA beads (Qiagen). All affinity purified proteins were further purified by Superdex 200 HR 10/30 gel-filtration using the AKTA purifier (GE Healthcare). Final preparations of purified proteins were checked by Coomassie staining of SDS polyacrylamide gels. Mammalian expression vectors for human Sirt2 and clover that contain entire coding region were ligated into the NheI and HindIII sites in pcDNA6/V5-His (Invitrogen). Sirt2-CTD and Clover-CTD expression vectors were generated by ligation of the C-terminal region of mouse Sirt1 (amino acid residues 511–734) into the pcDNA6 Sirt2 and Clover expression vectors by using the HindIII and XhoI sites. The Clover-ΔESA expression vector was constructed by sub-cloning the ΔESA Sirt1 CTD into the pcDNA6 Clover construct by using the HindIII and XhoI sites. All constructs were confirmed by DNA sequencing.

### Sirt1 deacetylase activity measurements

Biotin-conjugated histone H4 peptide corresponding to a.a. residues from 2 to 24 was radiolabelled by PCAF (P300/CBP-associated factor) with [^3^H]-Acetyl-CoA (MP Biochemicals-ICN, 3.7 Ci/mmole) for 6 h at 30 °C on a rotating platform. To maximize acetylation, an additional dose of fresh PCAF enzyme and [^3^H]-Acetyl-CoA were added into reaction mixture and incubated for additional 4 h. Acetylated peptide was captured by using streptavidin agarose beads and unbound peptide and free [^3^H]-Acetyl-CoA were removed by extensive washing with Tris-buffered saline buffer. Sirt1- or other Sirtuins-catalysed deacetylation reactions were carried out in the deacetylase assay buffer (50 mM HEPES at pH 7.0, 1 mM DTT, 10 mM MgCl_2_, 200 mM NaCl, protease inhibitor cocktail and phosphatase inhibitor cocktail) containing Streptavidin-bound histone H4 (75,000 c.p.m.), 4 μg enzyme, 0.5 mM NAD^+^ and the indicated concentrations of ATP in 100 μl reaction volume. The reaction mixture was incubated at 37 °C on a rotating platform (250 r.p.m.) to evenly disperse the beads for 45 min. The reaction was terminated by adding 150 μl of the quenching solution (87 μl of 37% HCl and 9.5 μl of glacial acetic acid in 2.7 ml of distilled water) to the reaction mixture and then vortexing. After centrifugation (16,000 *g*) for 2 min in a microcentrifuge, 100 μl of the supernatant was collected for scintillation counting to quantify the liberated *O*-[^3^H] acetyl-ADP-ribose. The Sirt1-dependent activity was then calculated by subtracting the c.p.m. obtained in reactions containing no NAD^+^. All reactions were carried out under steady state conditions.

### Cell culture, transfection, and reagents

Primary MEFS, and 293 HEK (ATCC CRL-1573) were grown in DMEM media supplemented with 10% fetal bovine serum and maintained in a tissue culture incubator containing 5% CO_2_. To induce severe energy-depletion, the media was replaced with glucose-free media containing 25 mM 2-DG for 2–5 h. To acetylate substrate proteins, p300 expression vector was co-transfected with expression vectors for transient transfection as indicated in figure legends by using Polyfect reagent (Qiagen) according to the manufacturer's protocol. Twenty-four hours later, each plate was divided into two plates and further cultured. Forty-eight hours post transfection, the cells were treated as indicated in the figure legends. When transiently expressing p53, we pretreated transfected cells with MG132 (50 μM, 30 min) to stabilize p53. The following antibodies and reagents were obtained from commercial sources: anti-FLAG antibody (F3165, Sigma, dilution 1:1,000), M2-agarose (A2220, Sigma, dilution 1:40), and V5-agarose (A7345, Sigma, dilution 1:40); anti-V5 antibody (R960-25, Invitrogen, dilution 1:5,000); anti-p53 antibody (DO-1, sc-126, Santa Cruz Biotechnology Inc., dilution 1:500), anti-p65/RelA antibody (sc-109, Santa Cruz Biotechnology Inc., dilution 1:1,000) and acetyl-Foxo1 antibody (D-19, sc-49437, Santa Cruz Biotechnolgy Inc., dilution 1:500), acetyl-p53 (K382) antibody (#2525, Cell Signaling Technology, dilution 1:1,000) and acetyl p65 (K310) antibody (D2S3J, #12629, Cell Signaling Technology, dilution 1:1,000); immobilized γ-Amino-hexyl-ATP agarose (Jena Bioscience, dilution 1:40). Uncropped scans of the most important blots were shown in Supplementary Figs. 4–8 in the Supplementary Information.

### ATP binding measurements using photoaffinity-labelling

Sirt1 and BSA (2 ug each) were incubated with 0.5 μCi 8-azidoadenosine 5′-triphosphate (Affinity photoprobes, LLC) on ice in the dark for 30 min in a buffer containing 30 mM HEPES, pH 7.0, 200 mM NaCl and 10 mM MgCl_2_ (Buffer A). After incubation, the samples were irradiated by using an ultraviolet stratalinker (Stratagene). During the exposure to ultraviolet radiation, the samples were placed on ice to prevent overheating. The exposure time for radioactive 8-azidoadenosine 5′-triphosphate was 12 s. The ultraviolet-irradiated samples were immediately added to SDS sample buffer containing β-mercaptoethanol (β-ME), subjected to SDS–PAGE and visualized by Coomassie staining and autoradiography. For photoaffinity-labelling in Fig. 2d, which used non-radioactive 8-azidoadenosine 5′-triphosphate, the ultraviolet radiation exposure time was increased to 20 min. For peptide labelling, 8 μl peptide (300 nmole ml^−1^) was added to buffer A plus 16 μg BSA in a total volume of 50 μl. After 12 s of ultraviolet-irradiation on ice, 30 μl of Streptavidin Agarose beads (Thermo scientific) in 0.1% β-ME was immediately added to samples and incubated on a rotating platform at 4 °C for 2 h. The incubated samples were loaded into Micro Bio-Spin Chromatography columns (Bio-Rad) and extensively washed with PBS containing 500 mM NaCl and 1% Triton X-100. After centrifugation to remove the residual solution, these columns were counted by using the LS 6500 multi-purpose scintillation counter (Beckman).

### Detecting deacetylation of p53 (K328) by immunoblotting

Recombinant His-tagged WT or mutant Sirt1 (0.4 μg) was incubated with acetylated GST-p53 (0.25 μg) (ref. 50) and 0.5 mM NAD^+^ in deacetylase buffer (50 mM HEPES at pH 7.0, 1 mM DTT, 10 mM MgCl_2_, 200 mM NaCl, protease inhibitor cocktail and phosphatase inhibitor cocktail (Roche)). The reaction mixtures were incubated at 37 °C for the indicated durations and stopped by addition of SDS sample loading buffer. The loaded amounts of Sirt1 and GST-p53 were visualized with Coomassie staining, Ponceau S staining or western blotting. Deacetylation of Ac-p53 (K382) by Sirt1 was detected by immunoblotting with antibody specific for acetylated-p53 (Cell Signaling)

### Sirt1 kinetics calculation

The non-competitive model defined by Cleland^58^ was used for the data fitting. The data were plotted as reciprocal initial velocity, 1/*V*_*o*_, versus reciprocal substrate concentration, 1/[*S*]. Nonlinear mixed-effects model fitting function in the programming language R^59^ (http://www.r-project.org) was used for data fitting against the double-reciprocal form of the non-competitive model equation (equation 2), where *V*_*o*_ is initial velocity, *V*_max_ is maximal velocity of a reaction, *K*_*m*_ is Michaelis constant, *K*_*is*_=[*E*][*I*]/[*EI*], *K*_*ii*_=[*ES*][*I*]/[*ESI*], [*S*] is substrate concentration, [*I*] is inhibitor concentration, [*E*] is enzyme concentration, [*EI*] is enzyme and inhibitor complex concentration, [*ESI*] is enzyme and substrate and inhibitor complex concentration. The catalytic constant, *k*_cat_ was derived by fitting to equation (3).


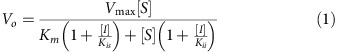



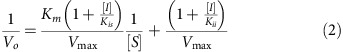



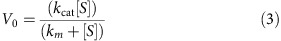


### ATP-binding measurements using gel-filtration

For generating the ATP-binding curve (Fig. 2e), we incubated full-length Sirt1 (2 μg) with 100 μCi [γ-^32^P]ATP (300 Ci/mmole, GE Healthcare) and non-radioactive ATP in assay buffer (30 mM HEPES, pH 7.0, 200 mM NaCl, 10 mM MgCl_2_, and 2 mM β-ME) in 20 μl final volume. Reaction mixtures were incubated on ice for 30 min and loaded onto Centri-Sep columns (Princeton separations), which were first hydrated with PBS. Unbound [γ-^32^P]ATP was removed by centrifugation, and bound [γ-^32^P ]ATP was measured by counting in a scintillation counter. Total ATP bound was calculated by the following formula: (bound cpm/input c.p.m.) × (radioactive ATP+non-radioactive ATP) for each concentration of ATP. The concentrations of bound ATP were plotted against the concentrations of input ATP and fitted to equation 4 using computer programming language R (http://www.r-project.org). The *k*_*d*_ value was determined using the nonlinear least squares fitting function of the programming language R.


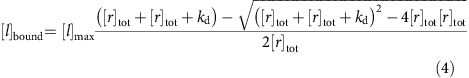


where [*l*]_bound_ is the concentration of bound ligand, [*l*]_max_ is the concentration of maximum bound ligand, [*r*]_tot_ is the total concentration of receptor, [*l*]_tot_ is the total concentration of ligand and *k*_d_ is the equilibrium dissociation constant.

### HPLC analysis of adenine nucleotides

A standard mortar and pestle was used to grind harvested cells or tissues in the presence of liquid nitrogen. Ground powders were dissolved in 5% perchloric acid and completely sonicated and centrifuged. The perchloric acid extracts were neutralized to pH 7 with a 3 M KOH and analysed by a modified ion-pairing high-performance liquid chromatography (HPLC)^60^. The Agilent 1,100 HPLC (Agilent technologies) was equipped with a reverse phase column, Supelco LC-18-T (150 × 4.6 mm, 3 mm, Supelco), the flow rate was 0.7 ml min^−1^ and detection was performed at 260 nm. The HPLC-reverse phase column was calibrated with AMP, ADP, ATP and NAD^+^ (Sigma-Aldrich).

### Transgenic mice

C57BL/6 and 1,29Sv mixed background Sirt1^flox/flox^ mice were obtained from Jackson Laboratory. Cardiac-specific (α-myosin heavy chain promoter-driven) Cre transgenic mice with C57BL/6 background, αMHC-Cre, were obtained from Dr Michael D. Schneider. Cardiac-specific Sirt1 knockout mice were generated by crossing αMHC-Cre with Sirt1^flox/flox^ mice.

### Ischaemia surgery

Male mice (2–7 months old) were anaesthetized by intraperitoneal injection of pentobarbital sodium (60 mg kg^−1^). A rodent ventilator (model 683; Harvard Apparatus Inc., Holliston, MA, USA) is used with 65% oxygen during the surgical procedure. The animals were kept warm using heat lamps and heating pads. Rectal temperature was monitored and maintained between 36.8 and 37.2 °C. The chest was opened by a horizontal incision through the muscle between the ribs (third intercostal space). Ischaemia was induced by ligating the anterior descending branch of the left coronary artery (LAD) using an 8–0 nylon suture, with a silicon tubing (1 mm OD) placed on top of the LAD, 2 mm below the border between left atrium and left ventricle (LV). After 24 h of ischaemia, the animals were anaesthetised and the chest was opened. KCL was injected at the diastolic phase to arrest the heart. The ascending aorta was cannulated and perfused with saline to wash out blood. The LAD was occluded with the same suture. Alcian blue dye (1%) was perfused into the aorta and coronary arteries. After excision of the hearts, Lversus were sliced into 1-mm-thick cross-sections. Those sections were incubated with a 1% triphenyltetrazolium chloride (TTC) solution at 37 °C for 10 min, and then incubated with 10% formalin for 4 h. The infarct area (pale discoloration), the area at risk, and the total LV area from both sides of each section were measured using ImageJ program, and the values obtained were averaged. The percentages of area of infarction and AAR of each section were multiplied by the weight of the section and then totalled from all sections. AAR/LV and infarct area/AAR were expressed as percentages. All procedures involved in live animals were performed in accordance with protocols approved by Rutgers Biomedical Health Science.

We chose our sample sizes based on those commonly used in this field without predetermination by statistical methods. The age- and weight-matched mice were randomly divided into each experimental group. The investigators were not blinded to the group allocation during experiments and outcome assessment.

### Generation of WT and mutant Sirt1 stable cell lines

To generate a lentiviral vector for Sirt1, full-length cDNAs of WT, HY and 2A mutant Sirt1 in pcDNA6/V5-His plasmids were digested with Nhe1 and Pme1 and cloned into Nhe1 and Swa1 sites of pCDH-GFP-Puro lentivirus vector (SBI). Lentivirus production and transduction into Sirt1 KO cells to make stably expressing cell lines were performed according to the instruction of the Virapower Lentiviral Expression system (Invitrogen).

### Adipogenesis

WT and 2A mutant Sirt1 cell lines were cultured in DMEM supplemented with 10% fetal bovine serum. Adipoyte differentiation was induced by treating confluent cells in differentiation media supplemented with insulin (Sigma) and triiodothyronine (T3, Sigma), followed by a 2-day incubation with induction media supplemented with insulin, triiodothyronine, indomethacin, dexamethasone and IBMX. Two days after induction, cells were cultured in differentiation media for 6 days. For Oil Red O staining, cells were fixed with 4% formalin and stained for 1 h with 0.5% Oil Red O solution in 70% isopropyl alcohol. Gene expression analysis was performed by SYBR Green PCR. Briefly, total RNA was isolated mini kit (Qiagen) and quantified by NanoDrop (NanoDrop Technology). cDNAs were synthesized using SuperScript cDNA synthesis kit (Invitrogen) and analysed by real-time PCR with SYBR Green method (Applied Biosystem). The relative quantity of each transcript was calculated by comparative Ct method normalized against *Gapdh*. The primers of target genes were purchased from MWG Operon and the sequences of primers were shown in Supplementary Table 4.

### Sedimentation velocity analytical ultracentrifugation

Sedimentation velocity experiments were conducted at 50,000 r.p.m. and 20 °C using a An50-Ti rotor on a Beckman Coulter ProteomeLab XL-I analytical ultracentrifuge following standard protocols^61^. Briefly, 10 μM samples of recombinant full-length Sirt1 in 150 mM NaCl, 40 mM Tris (pH 7.4), 20 mM MgCl_2_ and 0.5 mM TCEP was prepared. Sirt1 protein samples were also prepared at similar concentrations from the same stock solutions in 10 mM ATP or 10 mM NAD, by dilution of 100 mM ATP and 50 mM NAD-buffered stock solutions. All samples were loaded in two-channel centre-piece cells and analysed in the same sedimentation experiment with data collected using the Rayleigh interference (655 nm) optical detection system. Absorbance (280 nm) data were also collected for samples without ATP or NAD. Sedimentation data were time-corrected^62^ and analysed in SEDFIT 15.01c (ref. 63) in terms of a continuous c(*s*) distribution of Lamm equation solutions with a resolution of 0.05 S and a maximum entropy regularization confidence level of 0.68. Excellent data fits were observed with r.m.s.d. values ranging from 0.0041 to 0.0048 absorbance units and 0.0043 to 0.0071 fringes. All solution densities *ρ* and viscosities *η* were measured experimentally at 20 °C on an Anton Paar DMA 5000 density meter and Anton Paar AMVn automated rolling ball viscometer, respectively. Protein partial specific volumes were calculated based on the amino acid composition in SEDNTERP^64^ (http://sednterp.unh.edu/), and sedimentation coefficients *s* were corrected to *s*_*20,w*_ values at standard conditions.

### *In vivo* labelling of Sirt1 protein

Cells in the exponential phase of growth were harvested by treatment of trypsin and permeabilized to exogenously supplied [α-^32^P] ATP as described previously^65^ with some modifications. Cells were washed with PBS and treated on ice for 10 min with a hypotonic buffer (10 mM Tris-HCl (pH 7.8), 1 mM EDTA, 4 mM MgCl_2_, and 30 mM β-mercaptoethanol). The cells were centrifuged (3,000 *g* for 10 min) and resuspended in the same buffer (10^7^ cells per ml). These cells were added to a reaction mixture containing 33 mM Tris-HCl (pH 7.8), 20 mM β-ME, 0.6 mM EDTA, 42.5 mM MgCl_2_, 250 μCi of [α-^32^P] ATP (3,000 Ci/mmole, GE), 0.05% Triton X-100. After incubation for 1 h on ice, cells were collected and washed twice with cold PBS by centrifugation. The collected cells were lysed by using buffer containing PBS, 1% Triton X-100, protease inhibitor cocktail, and phosphatase inhibitor cocktail (Roche). After centrifugation (13,000 r.p.m. for 30 min at 4 °C), the supernatant was ultraviolet-irradiated on ice for 10 min and immunoprecipitated with Sirt1 antibody or where indicated, with Flag antibody-agarose (Sigma) beads. ATP bound Sirt1 protein was visualized by autoradiography and by immunoblotting.

### Statistics

Results are expressed as the mean±s.e.m. Comparisons between the treatment groups were analysed by two-tailed unpaired Student's *t*-test using GraphPad Prism 5 software (GraphPad Software). Differences were considered significant when *P*<0.05.

### Data availability

The authors declare that all the other data supporting the findings of this study are available within the article or Supplementary Information files. All other relevant data are available from the corresponding author upon request.

## Additional information

**How to cite this article:** Kang, H. *et al*. Sirt1 carboxyl-domain is an ATP-repressible domain that is transferrable to other proteins. *Nat. Commun.* **8,** 15560 doi: 10.1038/ncomms15560 (2017).

**Publisher's note:** Springer Nature remains neutral with regard to jurisdictional claims in published maps and institutional affiliations.

## Supplementary Material

Supplementary Information Supplementary Figures and Supplementary Tables
