# Supplementary material for: Sirt1 carboxyl-domain is an ATP-repressible domain that is transferrable to other proteins
Source: Nat Commun. 2017 May 15;8:15560. doi: 10.1038/ncomms15560 (PMC5440690; doi:10.1038/ncomms15560)
Supplement: Supplementary Information — Supplementary Figures and Supplementary Tables [file ncomms15560-s1.pdf]

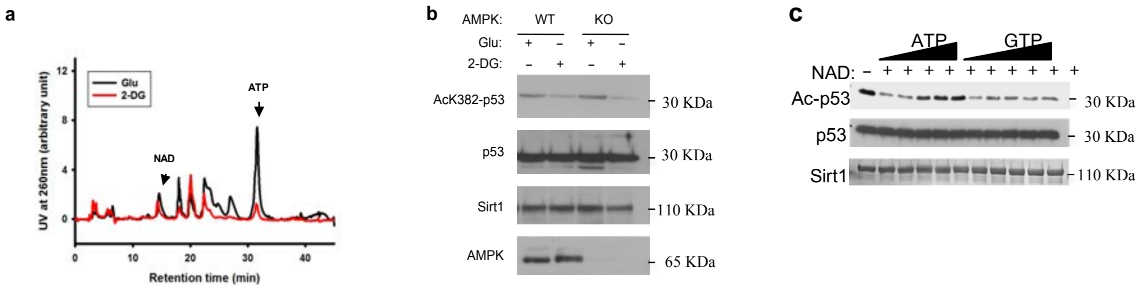

Supplementary Fig. 1. Sirt1 and energy depletion (**a**) Ion-pairing HPLC chromatogram of NAD^+ and ATP. 293 HEK cells were treated with either 25 mM Glu or 25 mM 2-DG for 2 hours. Cells were extracted and separated for quantitation as described in Experimental procedures. (**b**) Murine embryo fibroblasts derived from wild-type (WT) or AMPK α1α2 double knockout (KO) mice were incubated in media containing either glucose (25 mM) or 2-DG (25 mM) for 5 hours after ionizing radiation (20Gy). Acetylation of p53 was visualized by immunoblotting with antibody specific for Ac-k382. (**c**) Deacetylation reactions were performed with recombinant Sirt1 using Ac-p53 as substrate in the presence of 0-5 mM of ATP or GTP.

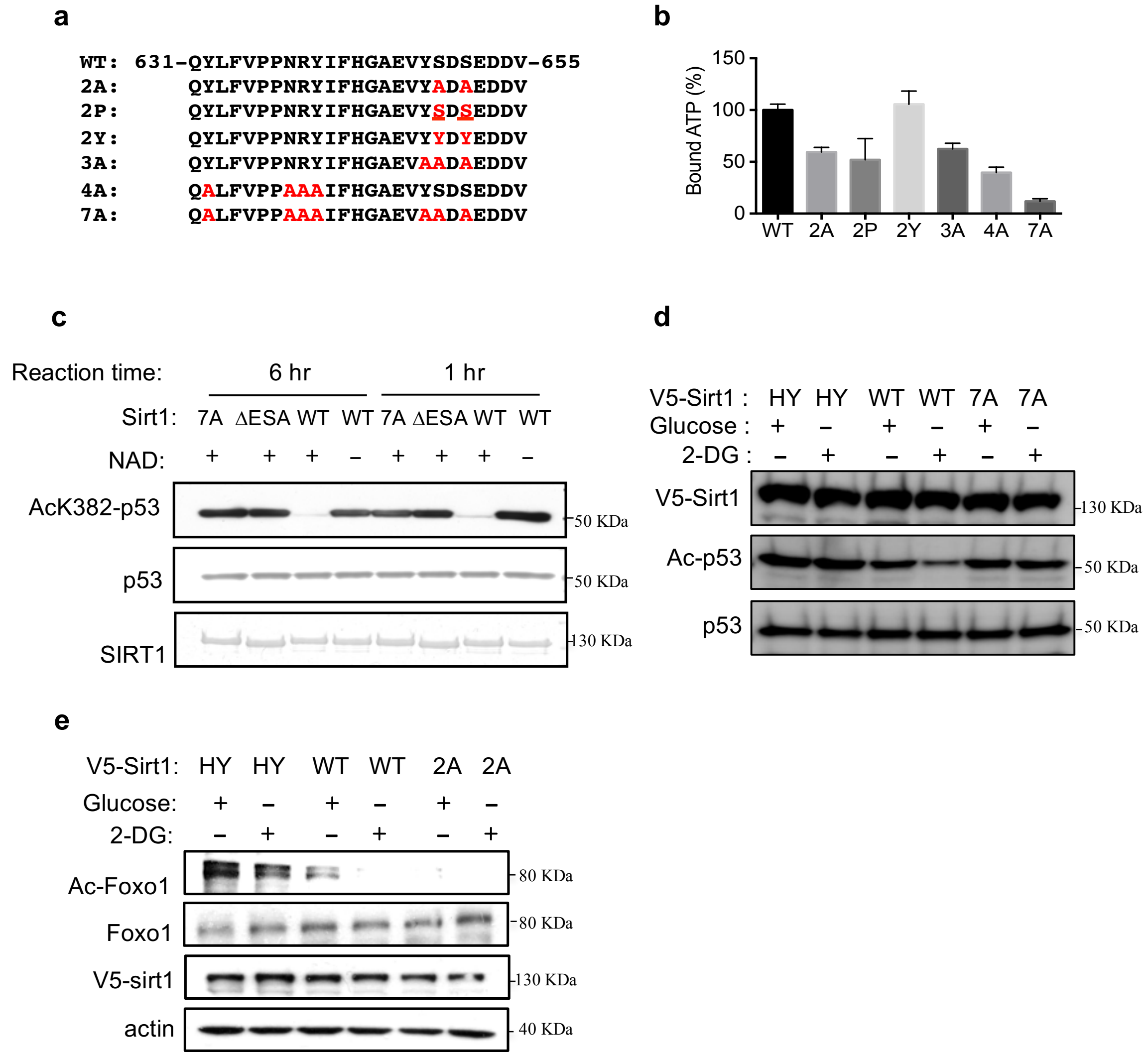

Supplementary Fig. 2. ATP binding to ESA peptide (**a**) The sequences of the ESA peptides used for ATP binding in panel B. The amino acid residues mutated to Ala(A), Tyr(Y) or phospho-Ser(S) in WT, A649/651 mutant (2A, 2Y, or 2P, respectively), A648/649/651 mutant (3A), A632/638/639/640 mutant (4A) and A632/638/639/640/648/649/651 mutant (7A) ESA peptides are indicated with red letters. (**b**) The relative levels of 8-azido-[α-^{32}P] ATP bound to these mutant ESA peptides compared with WT are shown (n=4). (**c**) Deacetylase reactions were performed with wild-type Sirt1 (WT) or Sirt1 without the ESA region (ΔESA) or with the 7A mutation using Ac-p53 as a substrate. Total levels of p53 and Sirt1 are shown below. (d) HEK293 cells were co-transfected with p53 expression vector and either HY, WT or 7A Sirt1 as indicated. The acetylation status of K382 in p53 was evaluated by immunoblotting with

an antibody specific for acetylated K382. (e) Sirt1 KO mefs stably expressing V5-tagged HY, WT or 2A Sirt1 were incubated with media containing either 25 mM Glu or 25 mM 2-DG for 2 hours and acetylation of Foxo1 was detected by immunoblotting whole cell extracts with acetyl Foxo1 antibody. A representative image from two independent experiments is shown.

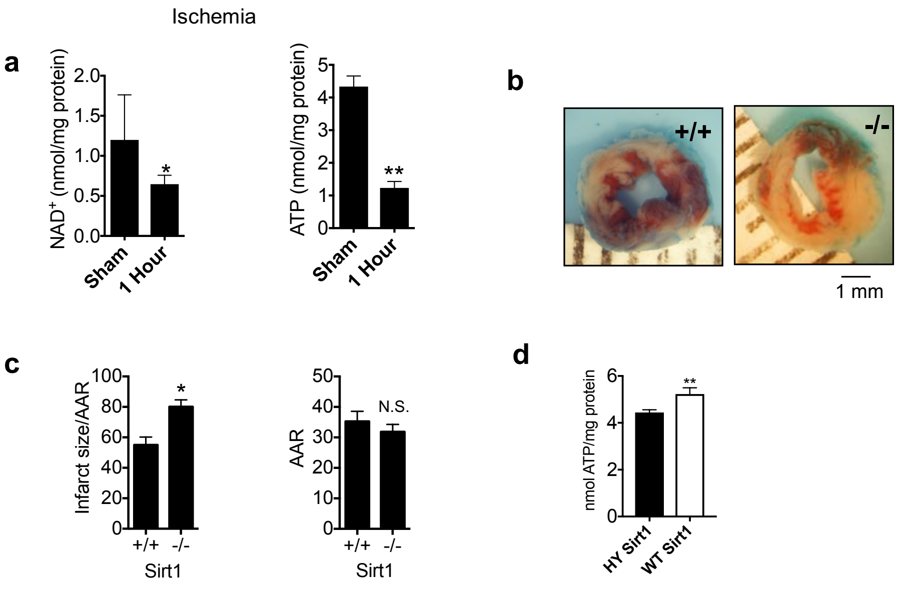

Supplementary Fig. 3. Sirt1 protects against ATP depletion-induced myocardial infarction (**a**) The levels of NAD^+ (left) and ATP (right) in sham and ischemic (1 hour) WT mice hearts were determined by HPLC. (**b**) Representative images for TTC staining of Sirt1-/- mice after 24 hours of ischemia. (**c**) The infarction area/area at risk (AAR) fraction was significantly greater in Sirt1-/- mice than in WT mice, while AAR was not significantly changed (N=5 for each group). (**d**) The levels of ATP in Sirt1 KO mefs stably expressing V5-tagged HY and WT Sirt1. Cells were incubated with DMEM media containing 10% fetal bovine serum (FBS) and harvested before reaching confluent state. Cells were extracted and separated for quantitation as described in Methods. Data are presented as mean ± SEM. *, $p<0.05$; **, $p<0.01$; N.S., not significant

Main Fig. 1a **Main Fig. 1b**

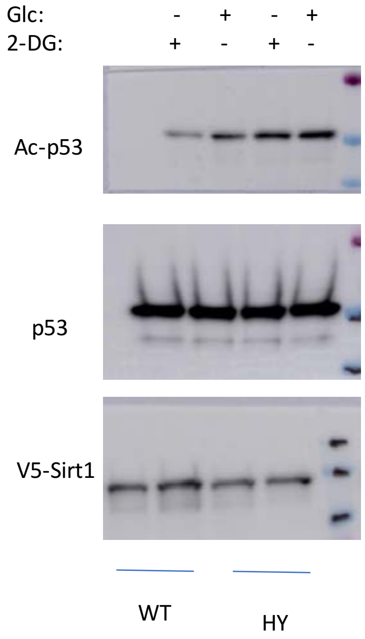

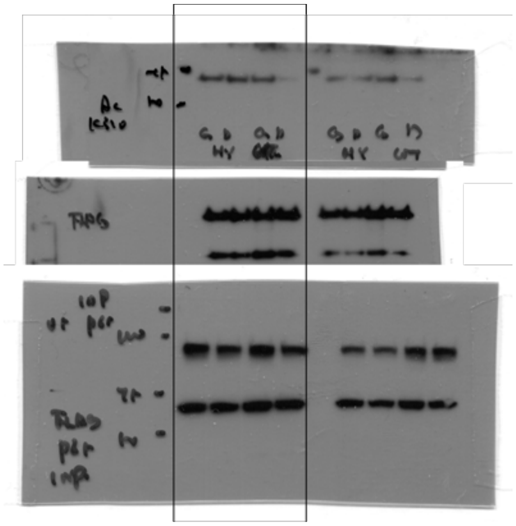

Main Fig. 1f

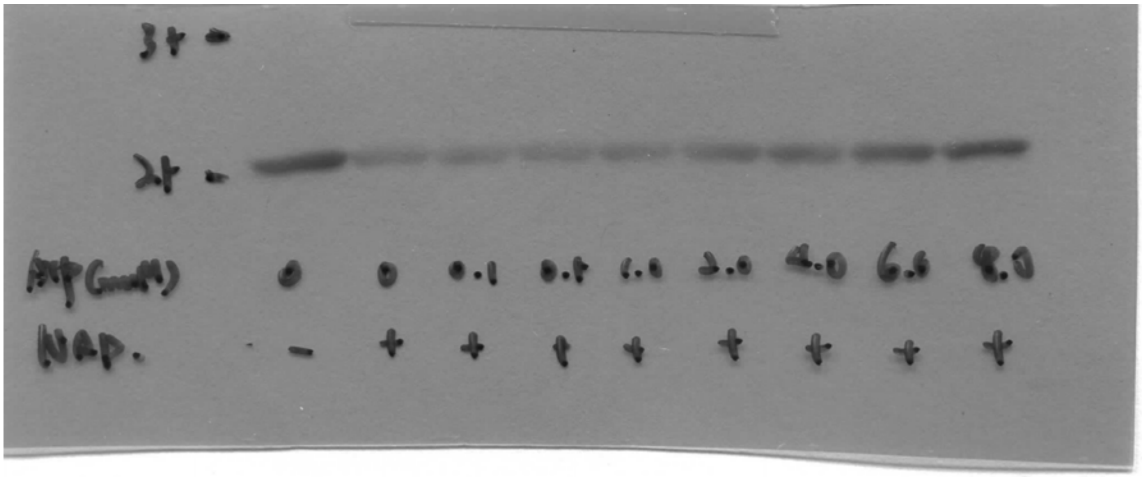

Supplementary Fig. 4. Uncropped scans of the most important Western blots presented in Fig. 1

Main Fig. 2a

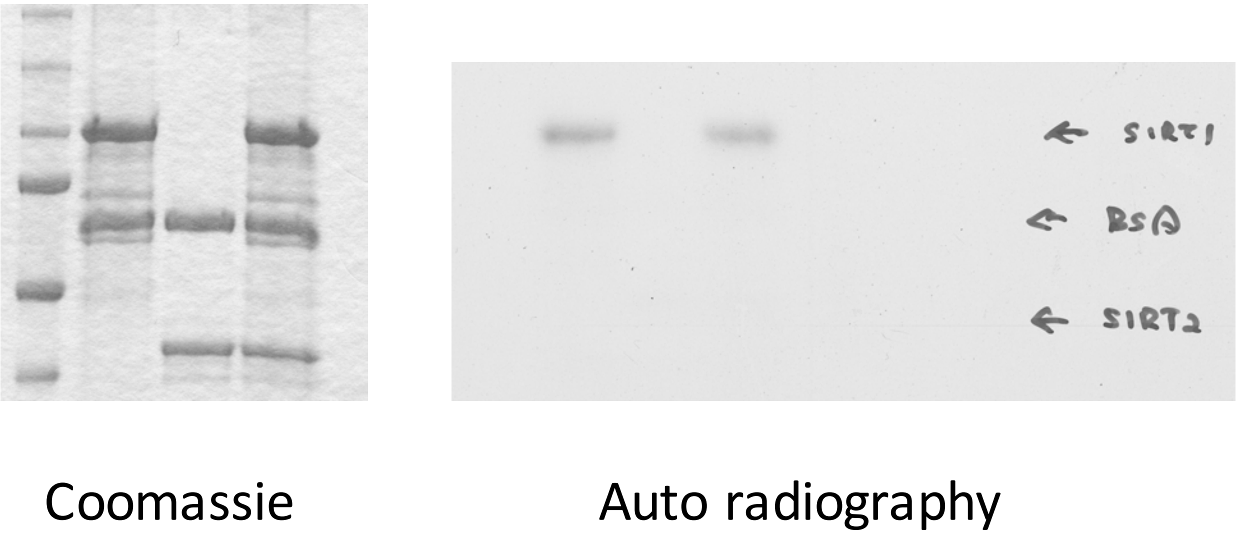

Main Fig. 2b

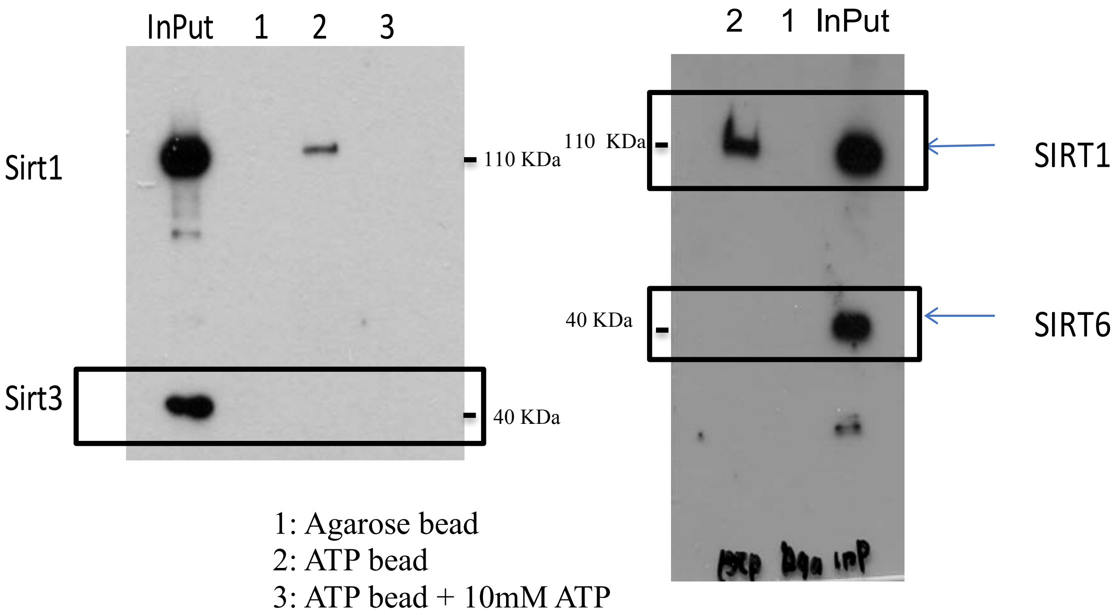

Main Fig. 2c **Main Fig. 2d** **Main Fig. 2i**

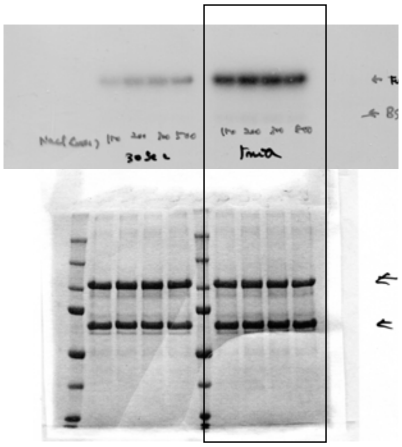

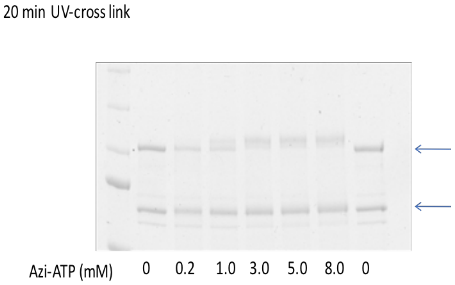

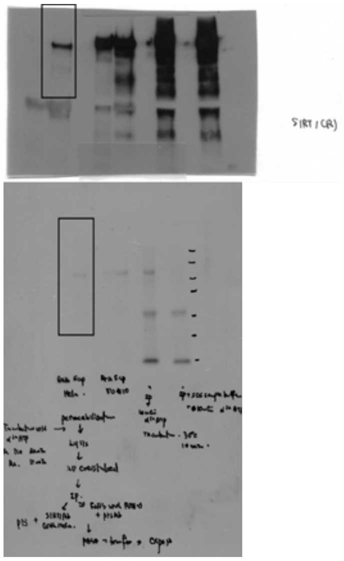

Supplementary Fig. 5. Uncropped scans of the most important Western blots presented in Fig. 2.

Main Fig. 3a **Main Fig. 4a.**

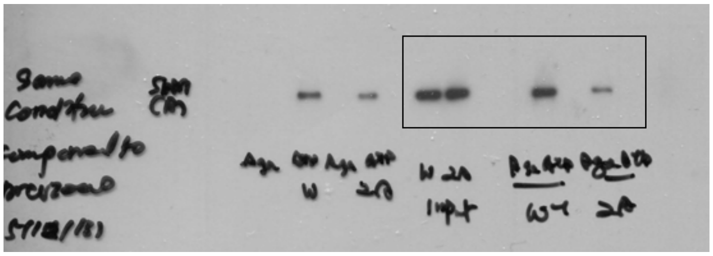

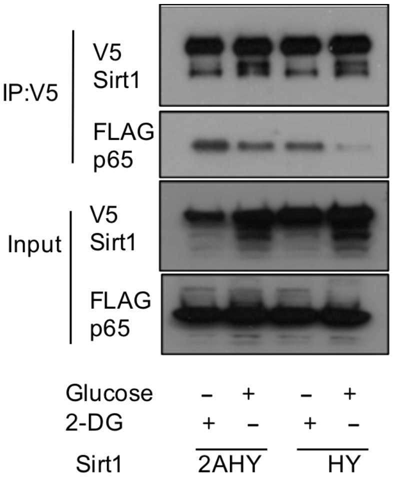

Supplementary Fig. 6. Uncropped scans of the most important Western blots presented in Fig. 3a and 4a.

Main Fig. 6a

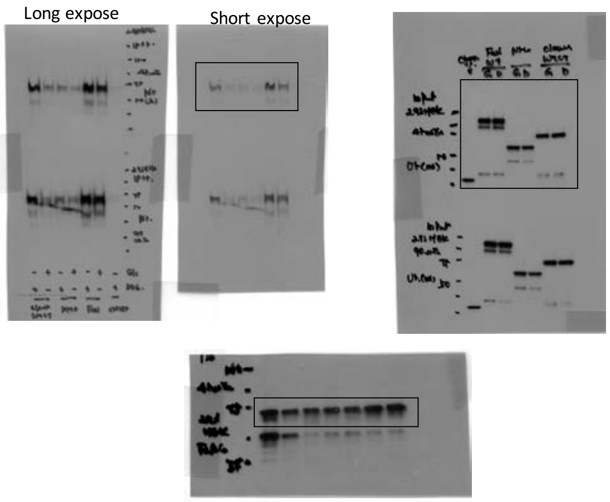

Main Fig. 6c

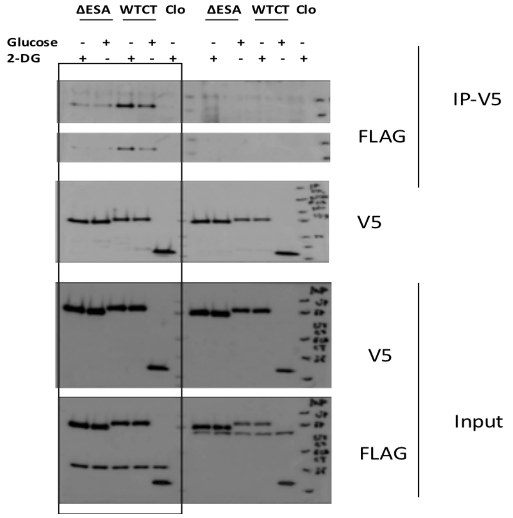

Main Fig. 6d

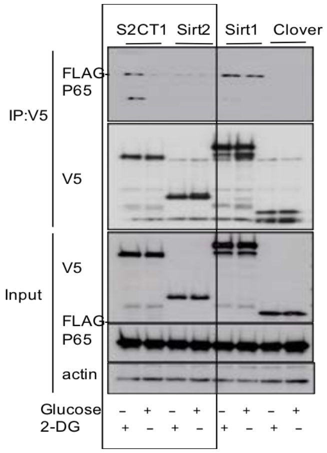

Main Fig. 6e

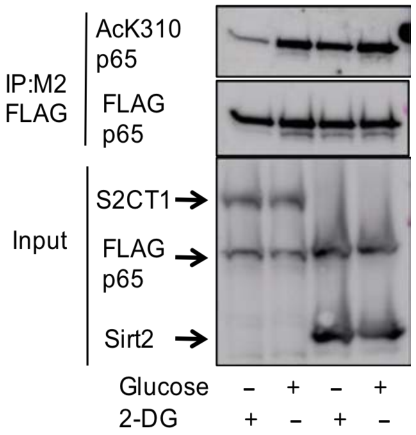

Main Fig. 6f

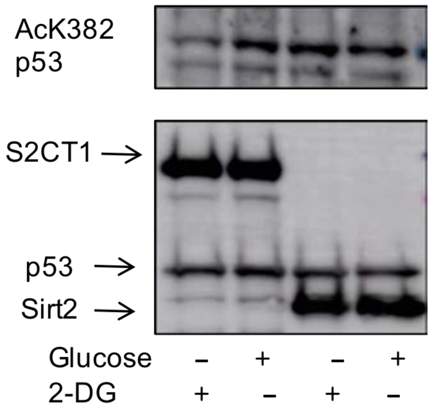

Supplementary Fig. 7. Uncropped scans of the most important Western blots presented in Fig. 6.

Supplementary Fig. 2c.

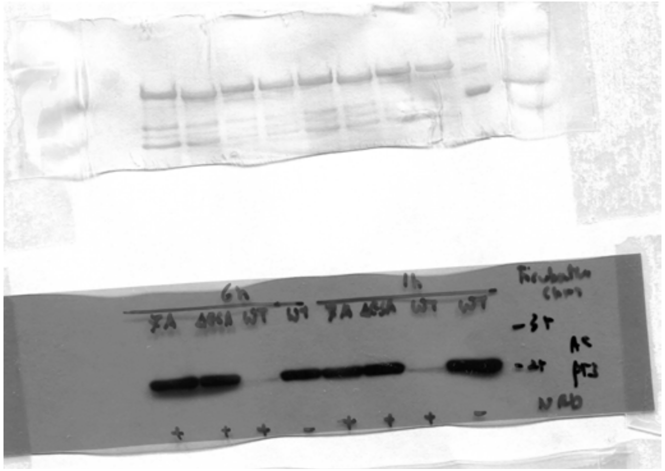

Supplementary Fig. 2d.

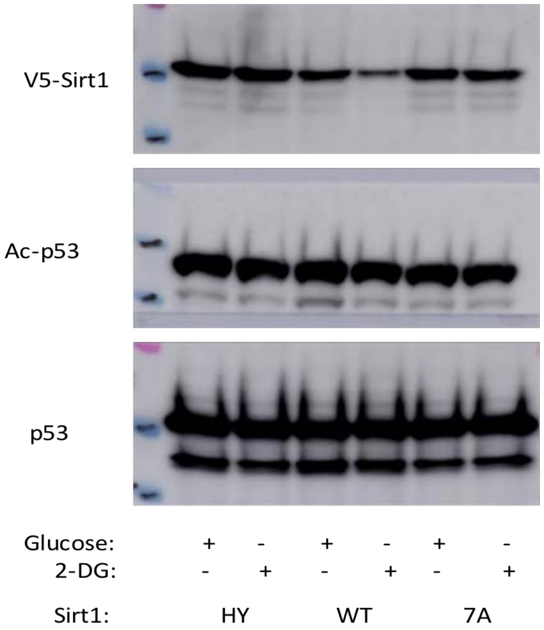

Supplementary Fig. 8. Uncropped scans of the most important Western blots presented in Supplementary Fig.2c and 2d.

Supplementary Table 1. ATP binding Sirt1 peptide sequence

Peptide Squence	Amino Acid #	Ion Score	Charge	m/z+ (Da)	MH+ (Da)	Mass Accuracy (ppm)
HGAEVYSDSEDDVL	643-656	36	2	768.332	1535.656	4.39
SDSEDDVLSSSSc*	649-661	46	2	694.265	1387.522	3.9
QSPSLEEPLEDESEIEEFY	671-689	78	2	1135.501	2269.994	3.1
YNGLEDDTERPEc*AGGSGF	689-707	38	2	1037.433	2073.859	6.46
NGLEDDTERPEc*AGGSGF	690-707	92	2	955.901	1910.794	6.36
c*AGGSGFGADGGDQEVVNEAIATR	701-724	51	3	780.019	2338.044	3.04
GADGGDQEVVNEAIATR	708-724	79	2	851.410	1701.812	4.78

High confidence peptide sequences were identified using the LTQ-Orbitrap MS/MS.

*Cystein residues that were found to be carbamidomethylated.

Supplementary Table 2. Summary of Kinetic Parameters

Enzyme	substrate	K_m (μM)	k_{cat} (min^{-1})
WT Sirt1	Ac-H4	26.83 ± 2.32	0.43 ± 0.08
WT Sirt1	NAD^+	203.08 ± 13.24	0.40 ± 0.04
2A Sirt1	Ac-H4	23.11 ± 0.80	0.41 ± 0.12
2A Sirt1	NAD^+	186.01 ± 7.07	0.54 ± 0.08

Supplementary Table 3. Inhibition constants for WT and 2A Sirt1

Enzyme	inhibitor	substrate	K_{ii} *(mM)*	K_{is} *(mM)*
WT Sirt1	ATP	Ac-H4	4.16 ±0.16	4.67 ± 3.01
WT Sirt1	ATP	NAD^+	3.00 ± 0.33	9.60 ± 2.61
2A Sirt1	ATP	Ac-H	6.45 ± 0.51	5.91 ± 7.23
2A Sirt1	ATP	NAD^+	4.35 ± 0.53	8.61 ± 1.38

Supplementary Table 4. The sequences of primers of target genes for SYBR Green PCR

Name	Primer sequence
aP2 (Fabp4)-Forward	5′-GATGCCTTTGTGGGAACCT-3′
aP2 (Fabp4)-Reverse	5′-CTGTCGTCTGCGGTGATTT-3′
Leptin-Forward	5′-CCTCATCAAGACCATTGTCACC-3′
Leptin-Reverse	5′-CCTCATCAAGACCATTGTCACC-3′
Adiponectin-Forward	5′-GCACTGGCAAGTTCTACTGCAA-3′
Adiponectin-Reverse	5′-GTAGGTGAAGAGAACGGCCTTGT-3′
Pparα-Forward	5′-GCGTACGGCAATGGCTTTAT-3′
Pparα-Reverse	5′-GAACGGCTTCCTCAGGTTCTT-3′
Pparγ-Forward	5′-TCAGCTCTGTGGACCTCTCC-3′
Pparγ-Reverse	5′-ACCCTTGCATCCTTCACAAG-3′
β-actin –Forward	5′-TTGCTGACAGGATGCAGAAG-3′
β-actin - Reverse	5′-GAAAGGGTGTAAAACGCAGC-3′
